# Structural basis for a human broadly neutralizing influenza A hemagglutinin stem-specific antibody including H17/18 subtypes

**DOI:** 10.1038/s41467-022-35236-y

**Published:** 2022-12-09

**Authors:** Yulu Chen, Fei Wang, Liwei Yin, Haihai Jiang, Xishan Lu, Yuhai Bi, Wei Zhang, Yi Shi, Roberto Burioni, Zhou Tong, Hao Song, Jianxun Qi, George F. Gao

**Affiliations:** 1 CAS Key Laboratory of Pathogen Microbiology and Immunology, Institute of Microbiology, Chinese Academy of Sciences, Beijing, 100101 China; 2 University of Chinese Academy of Sciences, Beijing, 100049 China; 3 Center for Influenza Research and Early-warning (CASCIRE), Chinese Academy of Sciences, Beijing, 100101 China; 4 Università Vita-Salute San Raffaele, Milano, 20132 Italy; 5 Research Network of Immunity and Health (RNIH), Beijing Institutes of Life Science, Chinese Academy of Sciences, Beijing, 100101 China

**Keywords:** Influenza virus, X-ray crystallography

## Abstract

Influenza infection continues are a persistent threat to public health. The identification and characterization of human broadly neutralizing antibodies can facilitate the development of antibody drugs and the design of universal influenza vaccines. Here, we present structural information for the human antibody PN-SIA28’s heterosubtypic binding of hemagglutinin (HA) from circulating and emerging potential influenza A viruses (IAVs). Aside from group 1 and 2 conventional IAV HAs, PN-SIA28 also inhibits membrane fusion mediated by bat-origin H17 and H18 HAs. Crystallographic analyses of Fab alone or in complex with H1, H14, and H18 HA proteins reveal that PN-SIA28 binds to a highly conserved epitope in the fusion domain of different HAs, with the same CDRHs but different CDRLs for different HAs tested, distinguishing it from other structurally characterized anti-stem antibodies. The binding characteristics of PN-SIA28 provides information to support the design of increasingly potent engineered antibodies, antiviral drugs, and/or universal influenza vaccines.

## Abstract (web summary)

Influenza continues to represent a major threat to public health, and the development of monoclonal antibodies (mAbs) that provide broad protection remains a key avenue for therapeutic development. Here, the authors demonstrate the molecular basis of neutralization for a human bnAb (PN-SIA28) against both endemic influenza A and emerging pathogens such as bat H17 and H18 viruses.

## Introduction

Influenza A virus (IAV), a genus of the *Orthomyxoviridae* family, remains a significant and persistent threat to human health and the world economy. Annual epidemics cause substantial morbidity and mortality, with 3–5 million cases of severe illness and 0.25–0.5 million deaths globally, and periodic influenza pandemics have the potential to kill millions^1–3^. Five influenza pandemics (1918 Spanish, 1957 Asian, 1968 Hong Kong, 1977 Russian, and 2009 swine flu) killed more than 40 million people since the beginning of the last century^4^. Furthermore, because of substantial influenza reservoirs in swine and avian populations^5^, the danger of a pandemic strain developing is always a concern. Several avian IAVs, including H7N9^6^, H7N4^7^, H5N1^8^, H5N6^9^, H5N8^10,11^, and H10N3^12^ have recently caused sporadic human infections.

In addition, the host species of IAVs have expanded to bats since the identification of H17N10 and H18N11 from bat samples^13,14^. Despite having similar genomes and being near to IAVs, both H17N10 and H18N11 viruses differ from known H1–H16 IAVs in a number of ways, including virus cultivation and replication, the cellular receptors exploited, and neuraminidase (NA) functions^13–15^. Their zoonotic potential remains unknown because they can use human MHC II molecules as an entry mediator^16,17^.

The current strategy to prevent influenza virus infection mainly relies on vaccination. However, due to the rapid evolution of influenza viruses, strain-specific vaccines that elicit antibodies (Abs) that neutralize vaccine strains and other homologous strains must be updated almost annually based on World Health Organization (WHO) surveillance and prediction of the strains likely to circulate in the coming year^18,19^. Thus, the inappropriate prediction of circulating strains can render the vaccine less effective. Antiviral drugs are another alternative for the treatment of influenza, especially in the very early stages of infection. Most notable are NA inhibitors that prevent the release of nascent virions^20,21^. Unfortunately, the widespread use of these drugs has resulted in the emergence of resistant viral strains without loss of transmissibility^22^. Given the emergence of the lack of cross-protective vaccines and anti-viral drug tolerance, there is an urgent need for the development of more effective antiviral drugs and therapeutic monoclonal Abs (mAbs) that can provide broad protective activities to prevent and treat influenza^23^.

Most Abs induced by vaccination or infection mainly target the hemagglutinin (HA) protein. The globular HA head domain recognizes host proteins bearing sialic acid on their surface, while the HA stem triggers the fusion of viral and host membranes following endocytosis, allowing release of viral contents into the cell^24^. Compared to the head domain, the stem region is less prone to mutations and relatively conserved across divergent influenza subtypes^25^. Anti-stem antibodies exhibit much broader cross-subtype neutralizing activity in vitro and in vivo by inhibiting the low pH-induced HA conformational rearrangement, hence blocking membrane fusion^24^. The majority of the known heterosubtypic stem binding antibodies neutralize IAV group 1 (H1, H2, H5, H6, H8, H9, H11, H12, H13, H16, H17, and H18)^26–29^ or group 2 (H3, H4, H7, H10, H14, and H15)^30–34^ subtypes. In general, anti-stem antibodies capable of recognizing both group 1 and 2 IAVs are extremely rare^35–39^. Such broadly neutralizing Abs hold great promise as potential broad-spectrum prophylactic or therapeutic agents and for the design of a universal influenza vaccine.

We previously reported a VH3-30-encoded mAb isolated from a patient exposed to circulating influenza A strains, with a detectable serum neutralizing activity against a 1934 influenza A isolate^40^. The mAb, named PN-SIA28, targets the HA stem region and neutralizes influenza A H1N1, H2N2, H5N1, and H9N2, as well as all H3N2 viruses from 1968 to 1975, in vitro^41^. In addition, PN-SIA28 can protect mice against A/WSN/33 (H1N1), A/Victoria/3/75 (H3N2), or A/Quebec/144147/09 (H1N1) pdm09-like viruses in vivo^42^.

In this work, we demonstrate that PN-SIA28 exhibits broader neutralizing activity in vitro and protects mice against H5N6 viruses in vivo. Besides group 1 and 2 conventional IAV HAs, PN-SIA28 can also inhibit bat H17 and H18 HA-mediated membrane fusion induced by low pH. Crystallographic analysis of Fab alone or in complex with H1, H14, and H18 HA proteins reveal that PN-SIA28 binds a highly conserved epitope in different HA stem regions with a unique binding modality. Our structural and biological data suggest the value of PN-SIA28 in treatment and vaccine development against IAVs.

## Results

### PN-SIA28 neutralizes divergent HA subtypes

**Fig. 1:**
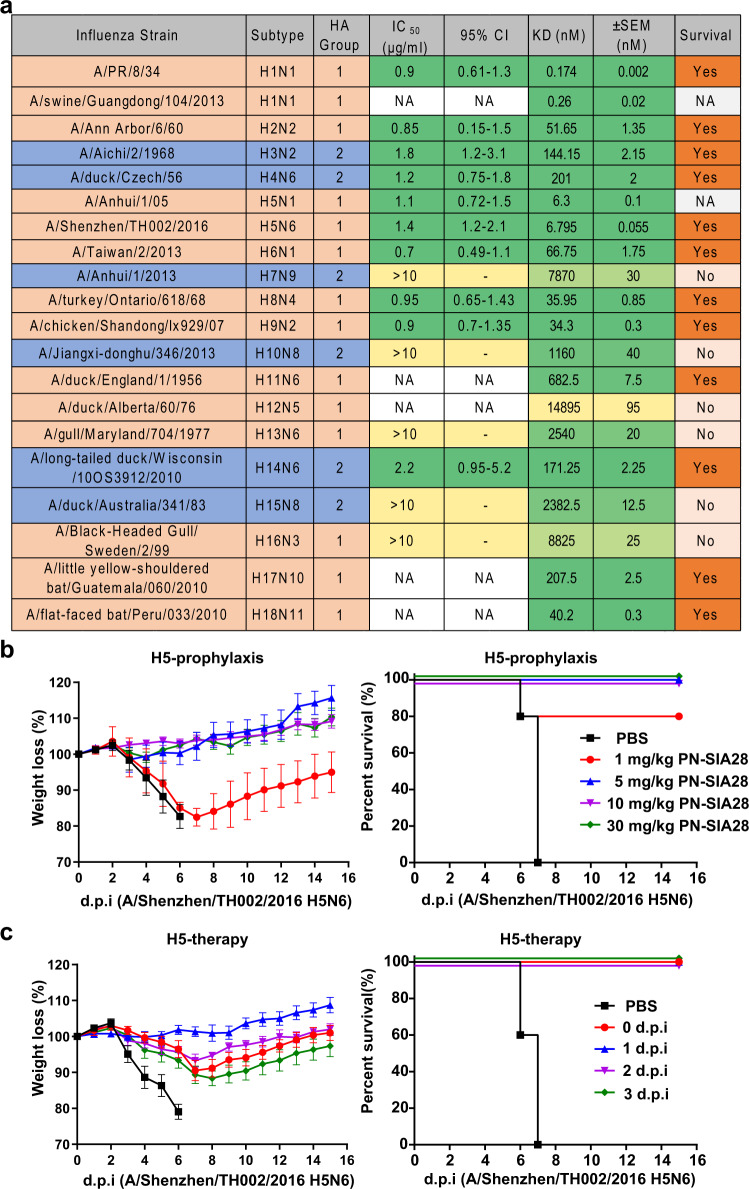
Broad reactivity and potent protective effect of PN-SIA28. **a** Binding and neutralizing activity of PN-SIA28. Binding kinetics were measured by BLI, and the K_D_ values shown are the mean ± SEM of two independent experiments. IC_50_, half-maximal inhibitory concentration; CI, confidence interval; NA, not available; survival, binding by gel filtration chromatography. Detailed binding properties of different HA proteins with PN-SIA28 are shown in Supplementary Fig. 2. **b** Prophylactic efficacy of PN-SIA28 against a lethal challenge with the A/Shenzhen/TH002/2016 (H5N6) virus. Shown are weight loss (left) and survival curves (right) of mice treated with 30, 10, 5, or 1 mg/kg of PN-SIA28 or PBS buffer 24 h before lethal challenge with an intranasal inoculation with the A/Shenzhen/TH002/2016 (H5N6) virus (at day 0). **c** Therapeutic efficacy of PN-SIA28 against a lethal challenge with the A/Shenzhen/TH002/2016(H5N6) virus. Shown are weight loss (left) and survival curves (right) of mice treated with PBS buffer (at day 1) or 15 mg/kg of PN-SIA28 right after or 1, 2, or 3 days after lethal challenge by an intranasal inoculation with the A/Shenzhen/TH002/2016 (H5N6) virus (at day 0) are shown. Error bars represent the mean ± s.d. (*n* = 5). Plots were generated using GraphPad Prism 7. Source data are provided as a Source Data file.

We previously identified a human mAb named PN-SIA28 from memory B cells isolated from the peripheral blood mononuclear cells (PBMCs) of a 55-year-old patient exposed to pre-2009 H1N1-pandemic influenza A strains, with a detectable serum-neutralizing activity against a 1934 influenza A isolate^40^. This mAb targets the conserved stem region of HA and has a broad neutralizing activity against several subtypes of IAVs^40^. PN-SIA28 had a large number of somatic mutations in both VH3-30*01 and VK1-12*01 genes compared to the unmutated common ancestor, and has a long heavy chain complementarity determining region 3 (CDRH3) (16 amino acids) (Supplementary Fig. 1). Preliminary neutralization assays show that PN-SIA28 neutralizes influenza A H1N1, H2N2, H5N1, and H9N2, as well as all H3N2 viruses from 1968 to 1975, in vitro^41^. However, we hypothesized that PN-SIA28 might have neutralizing activity against other IAV strains. To verify the cross-reactive breadth of PN-SIA28, we measured the binding of PN-SIA28 to a panel of recombinant HAs from both group 1 and group 2 IAVs by gel filtration chromatography. We found that PN-SIA28 bound 12 IAV subtypes (H1, H2, H3, H4, H5, H6, H8, H9, H11, H14, H17, and H18) out of the 18 HAs tested (Fig. 1a and Supplementary Fig. 2). Bio-layer interferometry (BLI) was then used to measure PN-SIA28’s affinity for all HA subtypes (H1-H18), and the results were generally consistent with gel filtration results (Fig. 1a and Supplementary Fig. 3). PN-SIA28 cross-reacted with all 18 subtype HAs, with high affinities (KD < 1 μM) to 12 HA subtypes. To further confirm the neutralization capability of PN-SIA28, divergent IAV strains were evaluated using an in vitro microneutralization assay. Consistent with the binding assays, PN-SIA28 neutralized H4N6, H6N1, and H14N6 strains with IC_50_s of 0.5-3 μg/ml. Additionally, PN-SIA28 also effectively neutralized two highly pathogenic avian influenza strains, H5N1 and H5N6, with IC_50_s of 1.0-1.5 μg/ml (Fig. 1a). These results suggest that PN-SIA28 has broad neutralizing activity against divergent group 1 and group 2 IAV strains.

### Protection efficacy of PN-SIA28 in vivo

Previous studies provide strong evidence of the protection conferred by the PN-SIA28 IgG molecule after a single dose administered 24 h after a lethal challenge with influenza A/WSN/33 (H1N1), A/Quebec/144147/09 (A[H1N1]pdm09), or A/Victoria/3/75 (H3N2) strains in mice^42^. In this work, we chose A/Shenzhen/TH002/2016 (H5N6) IAV for prophylactic and therapeutic assays in the BALB/c mouse challenge model. A/Shenzhen/TH002/2016 (H5N6) is a highly pathogenic strain that caused sporadic human infections in China^43^. In the prophylaxis assay, the mice were first given different doses of PN-SIA28 (30, 10, 5, or 1 mg/kg) and then challenged with a lethal dose of H5N6 virus 24 h later. Mice (100%) receiving PN-SIA28 at a high dose (≥5 mg/kg) survived challenge with A/Shenzhen/TH002/2016 (H5N6), and all displayed increases in body weight over 2 weeks (Fig. 1b). Only 80% of mice that received PN-SIA28 at a low dose (1 mg/kg) survived (Fig. 1b). In the therapeutic assay, the mice were first challenged with a lethal dose of the H5N6 virus and then treated with a fixed dose of PN-SIA28 (15 mg/kg) on different days (0, 1, 2, or 3 days). Surprisingly, 100% of the mice treated with 15 mg/kg of PN-SIA28, even up to 3 days after infection, were protected (Fig. 1c). Altogether, the results of prophylactic and therapeutic experiments indicate that PN-SIA28 effectively protected mice against the H5N6 virus.

### PN-SIA28 mechanisms of antiviral activity

**Fig. 2:**
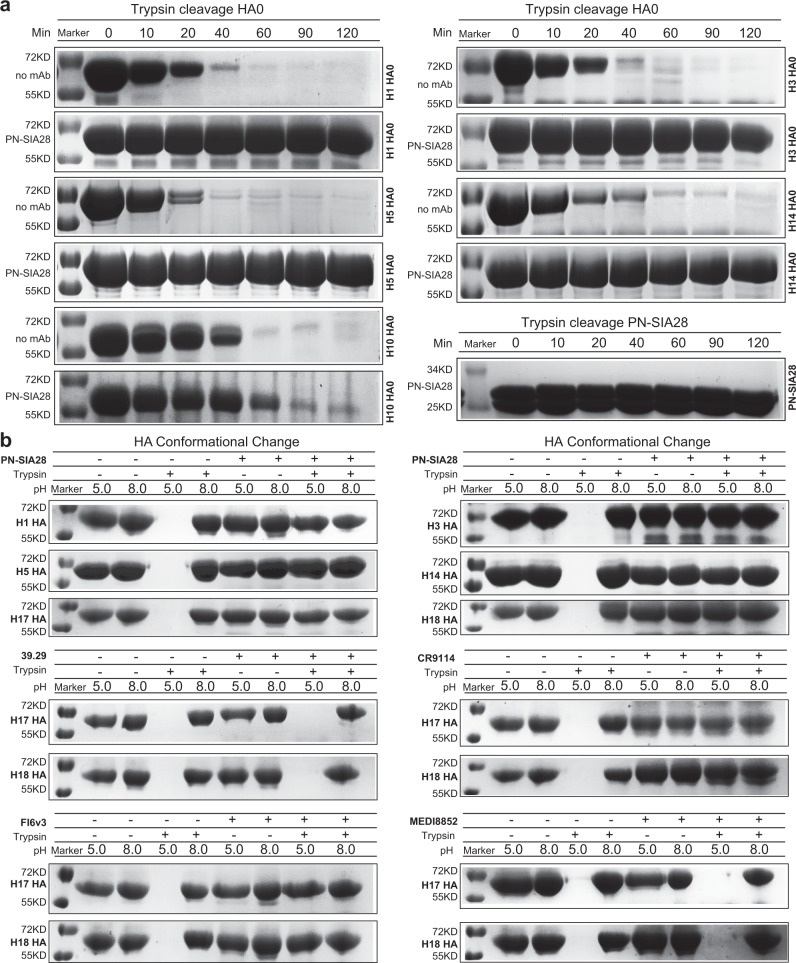
PN-SIA28 blocks proteolytic activation and inhibits low pH-induced conformational change in HA. **a** SDS–polyacrylamide gel electrophoresis results of uncleaved (HA0), recombinant-soluble H1, H5, H3, or H14 HA after digestion with trypsin at pH 8.0 for 0, 10, 20, 40, 60, 90, or 120 min. Digest reactions of HA with or without PN-SIA28 were stopped at several time points by adding a loading buffer containing SDS and dithiothreitol. H10 and trypsin-treated PN-SIA28 served as the experimental control and negative control, respectively. Data represent a representative experiment from two independent experiments. **b**, SDS–polyacrylamide gel electrophoresis results of the protease-susceptibility assay for HAs. Exposure of HA to low pH converts the HA to the protease-susceptible, post-fusion state (lane 3). Treatment of HA with PN-SIA28 before low-pH treatment blocks the pH-induced conformational change, retaining HAs (H1, H5, H3, H14, H17, and H18) in the protease-resistant, prefusion state (lane 7). Treatment of HA with CR9114 and FI6v3 before low-pH treatment blocks the pH-induced conformational change, retaining HAs (H17 and H18) in the protease-resistant, prefusion state (lane 7). In contrast, the pH-induced conformational change of H17 and H18 could not be blocked by treatment of HA with 39.29 or MEDI8852 prior to low-pH treatment. Data represent a representative experiment from two independent experiments. Source data are provided as a Source Data file.

The cross-subtype neutralizing antibodies reported to date that target the stem region of HA function mainly function by inhibiting HA-mediated membrane fusion activity in vitro^31,35,39,44,45^. The cleavage of the precursor of HA (HA0), and exposure of the cleaved HA to the low pH of endosomes initiate membrane fusion. Upon exposure to low pH, the structure of HA2 undergoes extensive conformational rearrangements, transforming from the pre-fusion state to the post-fusion state and bringing the viral membrane and the target endosomal membrane into close proximity to trigger membrane fusion^24,46–48^. Thus, PN-SIA28 is poised to inhibit these processes, which is illustrated by blocking HA0 maturation to HA and by decreased protease sensitivity of HA at low pH in the presence of PN-SIA28. In assays for these processes, we demonstrated that PN-SIA28 inhibits the host cell protease cleavage of both group 1 HA (H1 and H5) and group 2 HA (H3 and H14) HA0 molecules, which would prevent membrane fusion (Fig. 2a). Further, binding of PN-SIA28 to the cleaved HA (H1, H5, H3, or H14) also prevented its low pH-induced conformational change from the pre-fusion state to the post-fusion state (Fig. 2b). Notably, binding of PN-SIA28 to bat H17 and H18 HAs also prevented their low pH-induced conformational change, indicating the potential neutralizing activity of this mAb against bat H17N10 and H18N11 viruses. Like PN-SIA28, CR9114 and FI6v3 also prevented the low pH-induced conformational change of H17 and H18 HAs, but 39.29 and MEDI8852 did not have the same inhibitory effect on bat HAs, implying the distinct efficiencies for different antibodies (Fig. 2b).

### The structures of complexes formed between PN-SIA28 and H1, H14, or H18 HA

**Fig. 3:**
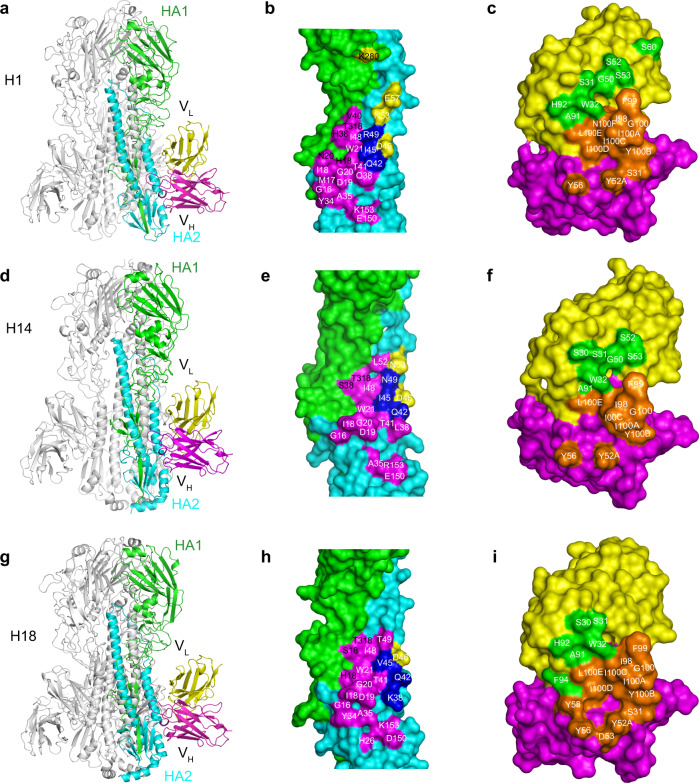
Structures of the PN-SIA28/H1, PN-SIA28/H14, and PN-SIA28/H18 complexes. The overall structures of the PN-SIA28/H1 (**a**), PN-SIA28/H14 (**d**), and PN-SIA28/H18 (**g**) complexes are displayed in cartoon representation. The PN-SIA28 Ab binds the conserved stem regions of the H1, H14, and H18 HAs. The epitope residues of PN-SIA28 in HA1 and HA2 of H1 (**b**), H14 (**e**), and H18 (**h**) are denoted in black and white characters, respectively. Residues of HA that are in contact with the heavy chain of PN-SIA28 are colored magenta, residues that are in contact with the light chain of PN-SIA28 are colored yellow, and residues that are in contact with both chains of PN-SIA28 are colored blue (**b, e, h**). The residues of PN-SIA28 responsible for the HA binding in the PN-SIA28/HA complexes (**c, f, i**) are marked in white characters. The heavy chain is colored magenta, and the light chain is colored yellow. The residues contacting the HA are colored orange for the heavy chain and colored pink for the light chain (**c, f, i**).

**Fig. 4:**
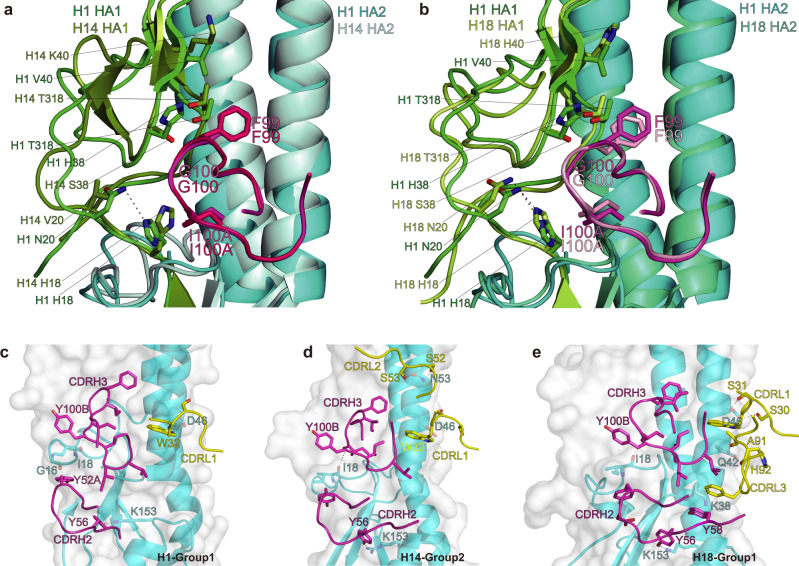
PN-SIA28 binds to different HA proteins with a unique binding modality. **a-b** PN-SIA28 interactions with HA1 of H1, H14, and H18. (**a**) Overlay of PN-SIA28 bound to group 1 (H1) and group 2 (H14) HA proteins. HA1 and HA2 of H1 are colored green and cyan, and HA1 and HA2 of H14 are colored split pea and pale cyan, respectively. The CDRH3 of PN-SIA28 binding to H1 is colored magenta, and the CDRH3 of PN-SIA28 binding to H14 is colored hot pink. (**b**) Overlay of PN-SIA28 bound to group 1 (H1) and group 2 (H18) HA proteins. The components of the PN-SIA28/H1 HA complex are colored according to (**a**). HA1 and HA2 of H18 are colored lemon and green-cyan, respectively. The CDRH3 of PN-SIA28 binding to H18 is colored light pink. The key amino acids that are in contact with the CDRH3 of PN-SIA28 are shown in stick representation. **c**–**e**, PN-SIA28 interactions with HA2 of H1 (**c**), H14 (**d**), and H18 (**e**). The main CDR loops of PN-SIA28 that are in contact with HA are shown in cartoon representation, and the heavy chain and light chain of PN-SIA28 are colored magenta and yellow. HAs are shown in surface and cartoon representation. Only the HA residues that form hydrogen bonds with PN-SIA28 are labeled and shown in stick representation. Polar contacts are drawn as dashed lines.

To provide insight into the structural basis of the broad neutralizing activity of PN-SIA28 against group 1 and 2 IAVs, PN-SIA28 Fab fragments or Fab variable fragments were prepared and co-crystallized with H1-HA (A/swine/Guangdong/104/2013), H14-HA (A/long-tailed duck/Wisconsin/10OS3912/2010), or H18-HA (A/flat-faced bat/Peru/033/2010). We determined the structures of the PN-SIA28 Fab fragment alone at 2.5 Å and of its Fab variable fragment complexes with H1, H14, or H18 HA at 3.2, 3.4, and 2.6 Å resolution, respectively (Supplementary Table 1, Supplementary Fig. 4). In our structures, we found that each Fab variable fragment interacts with just one protomer of the HA trimer, and PN-SIA28 recognized conserved residues in the stem region of HA by binding in a very similar orientation to the HAs. Overall, PN-SIA28 uses both its heavy and light chains to contact HA and bury approximately 1750, 1680, and 1646 Å^2^ from solvent for the H1, H14, and H18 complexes, respectively. The heavy chain of PN-SIA28 binds to the fusion subdomain of HA largely through an extended hydrophobic CDRH3 loop that inserts into a shallow hydrophobic groove between helix A of HA2 and the fusion domain component of HA1, whereas the light chain mainly interacts with the N-terminal region of helix A of HA2 (Figs. 3, 4). The epitope residues contacted by the PN-SIA28 heavy chain and light chain are quite similar between these three subtypes (Fig. 3b, e, h and Fig. 4). The heavy-chain paratopes or antigen-binding sites of PN-SIA28 in the HA complexes are remarkably similar, whereas the light-chain paratopes or antigen-binding site responsible for HA binding are obviously different (Fig. 3c, f, i). The heavy chain paratopes of PN-SIA28 in the PN-SIA28/H1, PN-SIA28/H14, and PN-SIA28/H18 complexes are composed of large portions of CDRH2 and CDRH3, whereas the light chain paratope of PN-SIA28 in the PN-SIA28/H1 complex is composed of large portions of CDRL1. The light chain paratope of PN-SIA28 in the PN-SIA28/H14 complex is composed of large portions of CDRL1 and CDRL2, while the light chain paratope of PN-SIA28 in the PN-SIA28/H18 complex is composed of large portions of CDRL1 and CDRL3 (Fig. 4 and Supplementary Table 2a–c).

For the interaction between the heavy chain of PN-SIA28 and H1, CDRH3 makes extensive contacts with the bottom of a hydrophobic groove that is formed by residues H18, N20, H38, V40, K280, and T315 (all residues are in H3 numbering) from the fusion domain component of HA1, G16, I18, D19, G20, and W21 of the fusion peptide, and Q38, Q42, I45, D48, and R49 of helix A (Fig. 2b, c and Supplementary Table 2a).

Among these resides, F99 (CDRH3) (Kabat numbering) binds in a upper position, and I100A (CDRH3) binds in a lower position, in the nonpolar groove. Y52A (CDRH2) also makes hydrophobic contacts with residues I18 and D19 of the fusion peptide. Additionally, Y56 (CDRH2) interacts with residues Y34 and A35 located at the bottom of helix A, as well as with residue E150 of helix G (Fig. 3b, c and Supplementary Table 2a). In particular, Y52A (CDRH2) makes a hydrogen bond with G16 of the fusion peptide, and Y56 (CDRH2) makes a hydrogen bond with K153 (Fig. 4c).

For the interaction between the light chain of PN-SIA28 and H1, CDRL2 and CDRL3 form minor interactions with H1, and the CDRL2 loop contacts residues from helix A of HA2 on the fusion subdomain. S31 (CDRL1) interacts with residue D46 of helix A, and W32 (CDRL1) makes hydrophobic contacts with residues Q42, I45, and R49 of helix A (Fig. 3b, c and Supplementary Table 2a). Other than hydrophobic interactions, W32 (CDRL1) forms hydrogen bonds with D46 of helix A (Fig. 4c).

Consistent with the antibody’s broad activity against group 1 and group 2 influenza viruses, the epitope of PN-SIA28 is highly conserved among H1, H14, and H18 HAs (Fig. 3b, e, h and Supplementary Fig. 5). However, the substitution of Ser (H14 and H18) for His (H1) at HA1 amino acid 38 could potentially weaken the binding of PN-SIA28 (Fig. 4a and Supplementary Table 2a–c). Aside from the amino acid differences at position 38, the different orientations of His at HA1 amino acid 18 could also affect the overall energetics of binding between HA1 and the CDRH3 loop (Fig. 4b and Supplementary Table 2a-c). Both the imidazoles of H18 from H1 and H18 HA1, which form hydrogen bonds with N20 of HA1, interact with the CDRH3 of PN-SIA28. In contrast, the imidazole of H18 from H14 HA1 shears off, which does not make a hydrogen bond with V20 of HA1 and has no contact with the CDRH3 of PN-SIA28. This seems to be a common trait shared by group 2 HAs (Fig. 4b and Supplementary Table 2a-c). Additionally, only V40 of H1 HA1, but not H40 of H18 HA1 and K40 of H14 HA1, can form hydrophobic interactions with the CDRH3 of PN-SIA28. All of the above-mentioned interactions involving the amino acids of HA1 contribute to H1 being the HA with the highest affinity for PN-SIA28 (Fig. 1a).

Aside from the interaction between residues at HA1 and CDRH3 of PN-SIA28 in the F subdomain, the most striking difference among the three PN-SIA28/HA complexes involves the interaction between the light chain and helix A of HA2. In the H14 complex with PN-SIA28, the light chain uses its extra CDRL2 to contact helix A. Notably, S52 (CDRL2) and S53 (CDRL2) are positioned within the hydrogen bonding distance of helix A N53 (Fig. 4d). In the H18 complex with PN-SIA28, the light chain uses its extra CDRL3 to contact helix A. Additionally, S30 (CDRL1) and S31 (CDRL1) are in the hydrogen bonding distance of helix A D46, and A91 (CDRL3) and A92 (CDRL3) are in hydrogen bonding distance of helix A Q42 (Fig. 4e). Moreover, the major epitope amino acids of the PN-SIA28 light chain on H1 (Q42, I45, and D46), H14 (Q42, I45, D46, and N53) and H18 (Q42, V45, and D46) are fairly conserved among the different HA subtypes (Supplementary Fig. 5).

### Comparison of PN-SIA28 with other broadly neutralizing antibodies

**Fig. 5:**
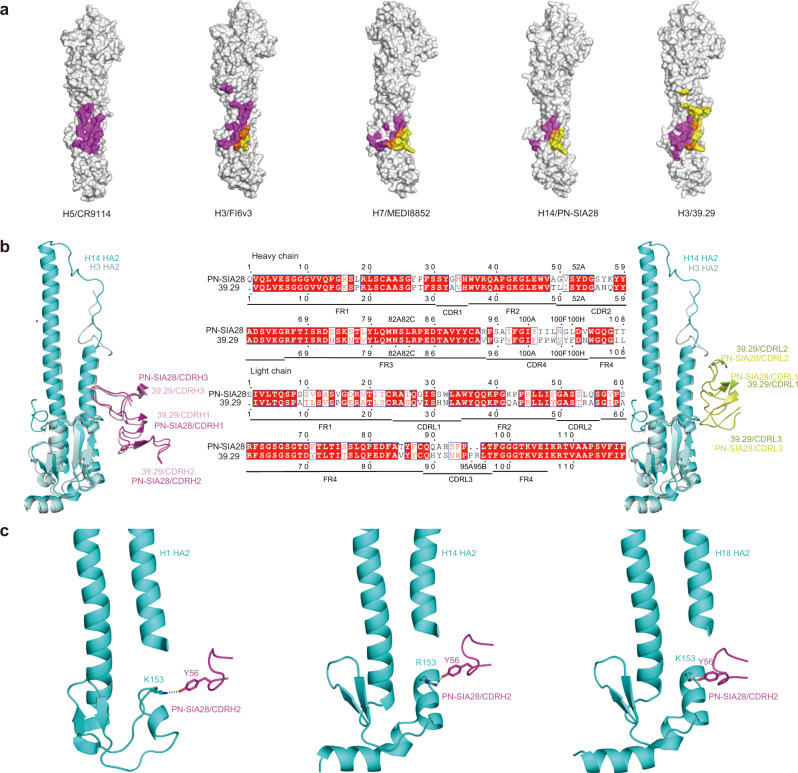
Binding specificities of PN-SIA28 compared to other stem-bound antibodies. **a** Epitopes of different broadly neutralizing antibodies on the HA surface. Residues of HA contacted by the heavy chain are colored magenta, residues of HA contacted by the light chain are colored yellow, and residues of HA contacted by both chains are colored orange. **b** PN-SIA28 interactions with H14 and comparison to the 39.29 interaction with H3. Overlay of PN-SIA28 bound to H14 and 39.29 bound to H3. HA2 of H14 and H3 are colored cyan and pale cyan. The heavy chains of PN-SIA28 and 39.29 are colored magenta and pink (left), and the light chains of PN-SIA28 and 39.29 are colored yellow and lemon (right), respectively. Alignment of VH and VL amino acid sequences of PN-SIA28 and 39.29 (middle). The amino acid residues are numbered (Kabat numbering), and the CDR segments are labeled. The conserved residues are in red. **c** Detailed views of PN-SIA28 interactions with H1, H14, and H18. The key interacting residues are shown as sticks, and polar contacts are drawn as dashed lines.

Comparison of the PN-SIA28, CR9114, FI6v3, MEDI8852, and 39.29 structures in complex with HA revealed that these five antibodies all recognize helix A of HA2 and the adjacent hydrophobic groove^35–37,39^. Although the epitopes of these antibodies on HA overlap extensively, the features of the interactions are markedly different. CR9114 only uses its heavy chain to bind HA, but the light chains of the FI6v3, MEDI8852, PN-SIA28, and 39.29 Abs account for 20, 30, 35, and 60% of the total buried surface area, respectively (Fig. 5a). This indicates that the light chain can play an important role in neutralization by broad-spectrum influenza antibodies, as we proposed earlier^34^. Compared to other broad-spectrum influenza antibodies targeting the stem region of HA, the most distinctive feature of PN-SIA28 is that it mainly uses the same CDRHs and different CDRLs to bind different HAs (including HAs of group 1 and 2) (Fig. 4c–e). However, other broadly neutralizing Ab (bnAb), such as FI6v3 and MEDI8852 mainly use the same CDRHs and the same CDRLs to bind different HAs of different groups (Supplementary Fig. 6).

In addition, PN-SIA28 and 39.29 both use the IGHV3-30*01 germline that has a long heavy chain complementarity determining region 3 (CDRH3) (16 amino acids). The comparison of the PN-SIA28 and 39.29 HA crystal structures shows that the heavy chains of PN-SIA28 and 39.29 adopt nearly the same topographical position on HA, and the light chains of PN-SIA28 and 39.29 display the same binding orientation (Fig. 5b). Another general feature of the PN-SIA28 complex with HA is the hydrogen bond between Y56 (CDRH2) and HA2 K153 (Fig. 5c and Fig. 4c, d, e). Sequence alignment of PN-SIA28 and 39.29 suggests that the substitution of Y56 (CDRH2) for N56 may be useful for 39.29 optimization (Fig. 5b, c).

### Conformational adaptation of PN-SIA28 upon complex formation

**Fig. 6:**
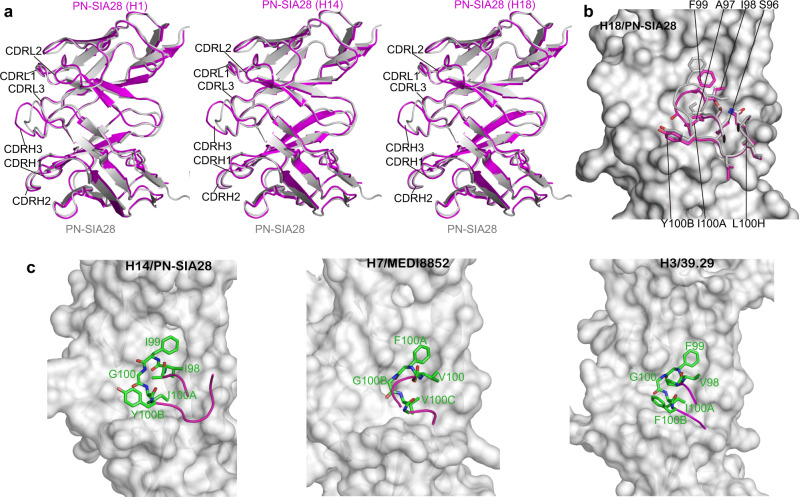
PN-SIA28 binds to a conserved stem region of HA through CDRH3 conformational rearrangements upon complex formation. **a** Overlay of PN-SIA28 bound to H1, H14, or H18 (magenta) and the PN-SIA28 Fab alone (grey). The CDRs of PN-SIA28 are labeled. **b** Conformational rearrangements in PN-SIA28 upon complex formation. Conformational change of the CDRH3 loops upon HA engagement. The CDRH3 of the apo and bound structures is colored gray and magenta, respectively. The beginning and end of the moving regions are indicated with black ovals. The HA is shown in surface representation. The apo structure does not make interactions with HA and does not fit into its surface features; the conformational change is necessary for productive HA engagement. **c** Comparison of the structures of the CDRH3 in the complexes between PN-SIA28/H14 (left panel), MEDI8852/H7 (middle panel), and 39.29/H3 HA (right panel). In all cases, the key amino acids are shown in stick representation with other loops of the antibody shown as coils, colored according to (**b**). The HAs are shown as gray surfaces.

The high-resolution structures of the PN-SIA28 Fab alone and PN-SIA28 Fab variable fragment in complex with H18 HA allow us to analyze conformational changes of PN-SIA28 upon HA binding in detail (Supplementary Table 1, Supplementary Fig. 4). The structural alignment of PN-SIA28 and PN-SIA28/H18 reveal that their CDR conformations are almost identical, except for CDRH3 (Fig. 6a). The CDRH3 loop formed by residues 97-100 G undergoes a largely rigid-body rotation, pivoted around S96 (CDRH3) and L100H (CDRH3) (Fig. 6b) to facilitate interactions with HA. Additionally, the side chain of F103 (CDRH3) moves by 4.5 Å to fit into the hydrophobic groove of the epitope.

As previously reported, 39.29 and MEDI8852 antibodies interact with residues in the hydrophobic groove and adjacent helix A in the fusion domain through the CDRH3 loop, which contains four similar amino acids (V-F-G-V/I)^37,39^. The corresponding amino acid sequence in the CDRH3 loop of PN-SIA28 is I-F-G-I (98-100 A). These tetrapeptides interact with the groove of their cognate HAs in a similar way (Fig. 6b). In addition, the amino acid at position 100B in the CDRH3 of PN-SIA28 is Tyr, which forms a hydrogen bond with I18 in the fusion peptide of different HAs (H1, H14, and H18) (Fig. 4c–e).

## Discussion

There is an urgent medical need for the isolation and identification of broadly neutralizing human mAbs against multiple influenza virus subtypes and novel subtypes of zoonotic influenza viruses from domestic animals in the treatment of severe influenza^49,50^. The potential of broadly infectivity-neutralizing antibodies targeting the conserved stem region of HA has provided insights into antibody-based therapy for severe and late-stage influenza, particularly for infants, the elderly, and immuno-compromised people. In addition, the characteristics of the epitopes and paratopes obtained by the analysis of antibody-HA complexes can be employed to guide the rational design of more effective therapeutic molecules and universal vaccines against harmful and potentially harmful influenza viruses^49^.

In this study, we performed structural and functional studies of a fully human mAb named PN-SIA28 that reacted with 12 HAs out of 18 influenza A antigenic subtypes and exhibited neutralizing activity against group 1 and 2 IAVs. Specifically, PN-SIA28 protects mice challenged with the H1N1 and H3N2 viruses^42^ and blocked infection and lethality of mice caused by the highly pathogenic H5N6 virus when administered up to 3 days after infection in vivo (Fig. 1a, b).

The discovery of bat influenza viruses has altered and broadened our understanding of IAVs. On a phylogenetic level, the bat IAV H17 and H18 HA proteins belong to group 1 HA subtypes rather than group 2 and share a notable degree of structural similarity with conventional IAV HA glycoproteins^15,51^. Furthermore, both H17 and H18 HAs exhibit typical HA characteristics, including harboring a monobasic proteolytic cleavage site and triggering syncytia formation at low pH (pH 5.4 and pH 5.6, which is within the normal pH range of most common HAs)^52^. The HA of bat H17N10 and H18N11 influenza viruses does not bind sialic acids because of the distorted putative sialic acid binding site^51^. The MHC class II DR protein, on the other hand, has been demonstrated to mediate cellular entry^16,17^. Furthermore, the H18N11 virus can enter 293 T cells when co-transfected with plasmids containing MHC II from bats, pigs, mice, or chickens^17^, implying its potential threat to humans. Two canine cell lines (MDCK II and RIE 1495) can support the replication of the bat influenza virus, suggesting that dogs may serve as intermediate hosts on the path to humans^53,54^.

Like other broad-spectrum Abs, the mechanisms of PN-SIA28-mediated neutralization of infection involve two processes of the infection cycle. Binding of PN-SIA28 to the stem of HAs on the infecting virus inhibits HA-mediated membrane fusion, which is critical for the initiation of infection. Additionally, PN-SIA28 prevented the formation and spread of newly made infectious virus through binding to precursor HA0 and blocking its cleavage at the end of infection (Fig. 2). Notably, in addition to conventional IAV HAs, PN-SIA28 could also inhibit H17- and H18-mediated membrane fusion, indicating its encouraging translational applications for both endemic and emerging potential IAVs.

The epitope recognized by PN-SIA28, confirmed by crystal structures of the PN-SIA28/HA complexes solved here, has previously been described for influenza group-specific antibodies and for more broadly reactive antibodies^35,37,39^. Comprehensive comparison of the structures of cross-group bnAb complexed with both group 1 and group 2 HAs helps to explain their broadly activities. The cross-group binding of FI6v3 (for H1 and H3) and MEDI8852 (for H5 and H7) is attributable to their identical flexible CDRs (CDRHs and CDRLs) recognizing highly conservative epitopes^35,39^. In stark contrast with these two bnAb, the binding of PN-SIA28 to different HAs involves the same CDRHs and different CDRLs. Given that the CDRL conformations in PN-SIA28 bound and unbound structures are almost the same and CDRLs in PN-SIA28/HA complexes only interact with Helix A of different HAs, the different Helix A orientations in different HAs cause them bind to different CDRLs (Supplementary Fig. 7). Like H14, the imidazole of H18 of HA1 from the group 2 HAs cannot form hydrogen bonds with V20 of HA1, thus preventing interaction with CDRH3s. This could be part of the structural basis for PN-SIA28 as a group 1-preferential neutralizing Ab. Furthermore, the carbohydrate side chain at N38 of HA1 may weaken the interaction between HA and PN-SIA28, which may explain why PN-SIA28 cannot efficiently bind to H7, H10, or H15. Altogether, the orientations of H18 of HA1 and Helix A of HA2 may help explain the cross-reactivity of distinct IAVs, including bat H17 and H18, despite the amino acid differences in the epitopes of HA.

Both PN-SIA28 and 39.29 use the IGHV3-30*01 germline^37^, but the substitution of Y56 for N56 in CDRH2 of PN-SIA28 increases the binding between the antibody and HA. Notably, PN-SIA28 neutralizes the H9N2 influenza A viruses, whereas 39.29 does not^39,41^. Additionally, the 39.29 light chain accounts for 60% of the total buried surface area compared to only 35% for PN-SIA28, which may explain why neutralization of 39.29 against the prevalent H3N2 virus in recent years is better than PN-SIA28^37,41^. The difference of neutralizing activity between PN-SIA28 and 39.29 may be mainly attributed to the light chain. The light chain of 39.29 interacts with more exposed regions of Helix A of HA2 than that of PN-SIA28. Therefore, the epitope amino acids of the 39.29 light chain are less conserved than those of PN-SIA28, contributing to the difference in the neutralization spectrum between 39.29 and PN-SIA28. Moreover, the PN-SIA28 heavy chain may bind to H3 HA by changing the orientation of the bulky carbohydrate chain attached to HA1 Asn38 like 39.29^37^. Altogether, this structural information helps to further optimize antibodies using the IGHV3-30*01 germline to produce more effective antibodies and underscores the necessity of developing proper heavy- and light-chain pairing. More importantly, this may represent a new mode of binding of cross-group neutralizing antibodies to different HAs.

The structural characterization of PN-SIA28 bound and unbound structures highlights its long and flexible CDRH3, which can accommodate the differences in conformation and environment in the hydrophobic grove of the HA. The comparison of the structures of the complexes formed by the Abs and HAs indicates that the three antibodies had the amino acid sequences, V-F-G-V-MEDI8852 (100-100 C), V-F-G-I- 39.29 (98-100 A), and I-F-G-I-PN-SIA28 (98-100 A) in their CDRH3 loops that occupied equivalent positions in the complexes^37,39^. Moreover, the Y100B in CDRH3 of PN-SIA28 forms a hydrogen bond with H1, H14 or H18 HA, and this may imply that the hydrogen bond may be a common interaction between PN-SIA28 and different HA subtypes. Conceivably, the structure of this pentapeptide (I-F-G-I-Y) of PN-SIA28 might be used to select candidate molecules on the basis of their affinity for the tetra-peptides or pentapeptides like P7^55,56^.

In conclusion, we show that PN-SIA28 is a bnAb for both endemic IAVs like H1, H3, and H5, as well as emerging potential IAVs, including bat H17 and H18. The structures shown here help us better understand cross-group heterosubtypic binding activity, which is significant in terms of the development of therapeutic countermeasures against IAV.

## Methods

### Viruses and cells

Viruses used in this study comprised wild-type isolates and reassortants, containing internal genes from A/Puerto Rico/8/1934, which were developed as candidate vaccine viruses for vaccine manufacturing (Fig. 1a). Viruses were propagated in Madin–Darby canine kidney (MDCK) cells or in embryonated eggs. For passive protection studies in mice, wild-type A/Shenzhen/TH002/2016 (H5N6) was amplified in embryonated eggs^43^. Virus titers were determined by end point dilution in MDCK cells (TCID_50_). MDCK cells were obtained from the American Type Culture Collection (Manassas, VA, USA) and cultured in Dulbecco’s modified Eagle’s medium (DMEM; Gibco, cat. no. 11965) with or without 10% fetal bovine serum (FBS). HEK293F cells were grown in suspension in SMM 293-TII medium (Sino Biological, Cat# M293TII) at 37 °C in a humidified 5% CO_2_ incubator rotating at 130 rpm. Sf9 and Hi5 insect cell line from Invitrogen were cultured at 27 °C. All cell lines were tested negative for mycoplasma contamination.

### Recombinant HA and Ab proteins

The gene fragments encoding the ectodomains of the HA proteins were individually cloned into the baculovirus shuttle vector pFastBac1(Invitrogen) by incorporating a GP67 signal peptide for HA secretion at the N-terminus and a thrombin cleavage site, a trimerization foldon sequence^57^, and a 6 × His tag at the extreme C-terminus for purification. The transfection and virus amplification were performed according to the user manual of the Bac-to-Bac Baculovirus Expression System (Invitrogen). Sf9 cells (Invitrogen) were used for virus amplification, and HA proteins were produced by infecting suspension cultures of Hi5 cells (Invitrogen) for 2 days and recovered from the culture supernatants by metal affinity chromatography using a 5-mL HisTrap column (GE Healthcare). The purified HA was subjected to thrombin digestion (Thermo, a maximum of 50 U/mg of HA0) at 4 °C for 12 h to remove the C-terminal trimerization foldon sequence and 6 × His tag. The HA0 was further purified by size exclusion chromatography using a Superdex 200 10/300 column, and then the collected protein fractions were concentrated to approximately 20 mg/mL in storage buffer (20 mM Tris, pH 8.0, and 150 mM NaCl) for use in structural and functional studies.

The gene fragments encoding the variable domains of the heavy chain and light chain of human mAb PN-SIA28 were cloned into the IgG1 heavy and light expression vectors, PCAGGS-H-hIgG1 and PCAGGS-L-hIgG1, respectively. HEK-293 F cells were transfected with the IgG1 expression plasmids, and the expressed antibody was purified from the culture supernatants using a Protein A column (GE Healthcare). PN-SIA28 Fab was obtained by digesting PN-SIA28 mAb with papain (30:1 mAb: papain ratio) for 8-10 h at 37 °C and purified by affinity chromatography using a Protein A column (GE Healthcare), followed by size exclusion chromatography using a Superdex 200 10/300 column (GE Healthcare). The purified PN-SIA28 Fab was concentrated to approximately 20 mg/mL in storage buffer (20 mM Tris, pH 8.0, and 150 mM NaCl) for use in structural studies.

The gene fragments encoding the variable domains of the heavy chain and light chain of human mAb PN-SIA28 were also cloned into the pET-21a expression plasmid. BL21 competent cells were transformed with the pET-21a expression plasmids, and the expressed scFV PN-SIA28 antibody was purified by inclusion body refolding^58^. The purified scFV PN-SIA28 was concentrated to approximately 10 mg/mL in storage buffer (20 mM Tris, pH 8.0, and 150 mM NaCl) for use in structural studies.

### Gel filtration survival detection

Recombinant HA proteins were mixed with purified Fab fragments at a molar ratio of 1:1.5 (HA: mAb) and incubated at 4 °C for 2 h in 20 mM Tris/HCl (pH 8.0), 150 mM NaCl buffer. The samples from individual HA reactions with or without Fab were then loaded onto a Superdex 200 10/300 GL column (GE Healthcare). The recorded chromatographs were overlaid, and the pooled proteins from each peak fraction were analyzed on 12% SDS-PAGE gels stained with Coomassie blue.

### Microneutralization assay

The microneutralization assays were performed as previously described^59^. Briefly, serial two-fold dilutions of mAb (1 mg/mL stock solution) in 50 mL were prepared and then mixed with the appropriate viruses (100 TCID_50_ in 50 mL per well). The mixture was placed in 96-well tissue culture plates and incubated for 1 h at 37 °C. Indicator MDCK cells (1.5 × 10^4^ cells/well) were added to each well and incubated at 37 °C for 24 h. To establish the endpoint, cell monolayers were then washed with PBS and fixed in acetone, and viral antigen was detected with an ELISA using a mAb anti-influenza A 7307 SPTN-5 against influenza A NP (Medix Biochemica) at a dilution of 1:5,000, and a HRP goat-anti-mouse IgG (H + L) secondary antibody (Invitrogen, 31430) at a dilution of 1:5,000. The virus neutralization titer was defined as the median of reciprocal values of the highest dilutions of antibody yielding ODs below the cutoff value. This cutoff is represented by a 50% specific signal calculated as (virus control OD + cell control OD)/2. The antibody concentration of the end point dilution (titer) represents the median neutralization of the virus analyzed.

### HA cleavage assay

The recombinant trimeric HAs from the H1N1, H5N6, H3N2, H14N6, H17N10, and H18N11 viruses were digested to HA1 and HA2 with TPCK-treated trypsin at a final concentration of 5 μg/mL for 2 h at 37 °C. The protease inhibitor Aprotinin (Sigma-Aldrich) was added to stop the reaction and then excluded by buffer exchange using a desalting column. Next, the protein was incubated with or without antibody (1:2 molar ratio) for 2 h at 37 °C. Then, the pH was lowered to 5.0 by replacing the buffer with 100 mM sodium acetate in all samples except for the controls. Being thoroughly mixed, the samples were incubated for 30 min at 37 °C. After incubation, the samples were equilibrated at room temperature, and the pH was neutralized by addition of 200 mM Tris (pH 8.5). TPCK-treated trypsin was then added to each sample to a final concentration of 10 μg/mL, and all samples were incubated at 37 °C for 4 h. The reaction was terminated by adding a loading buffer containing SDS. Samples were then loaded on a 12% SDS-PAGE gel after boiling at 95 °C for 5 min. H10 HA, which has low binding affinity for PN-SIA28, and trypsin-treated PN-SIA28 were acted as controls. To assess the ability of the Ab to block the cleavage of HA0 protein, baculovirus-expressed recombinant HA from A/PR/8/34 (H1N1) or A/Hong Kong/8/68 (H3N2) was incubated with or without PN-SIA28 at a molar ratio of 4:1 (mAb: HA) for 2 h. The HA with or without PN-SIA28 was then exposed to 2.5 µg/mL of TPCK-treated trypsin and further incubated for 10, 20, 40, 60, 90, and 120 min at 37 °C before being stopped by adding a loading buffer containing SDS and dithiothreitol. Samples were then loaded on 12% SDS-PAGE gels after boiling at 95 °C for 5 min.

### Binding affinity (K_D_) assay

The K_D_ of the HAs from different virus strains for the PN-SIA28 mAb was determined by BLI at 25 °C using an Octet Red96 instrument (ForteBio, Inc.). HAs were biotinylated and immobilized on SA-coated biosensor surfaces and then exposed to the PN-SIA28 fab in solution. HAs were loaded onto SA-coated biosensors in kinetics buffer (1x PBS, pH 7.4) for 300 s. To measure the binding affinities, the association and dissociation of HAs were measured by exposing the sensors to gradient concentrations of the PN-SIA28 fab in kinetics buffer. The kinetic data sets were fitted using a 1:1 binding model (Octet RED96 analysis software 7.0) to yield the K_D_.

### Protection effects of mAbs on influenza viruses

All animal studies for the evaluation of the protection effects of PN-SIA28 on influenza viruses were conducted in accordance with the protocol approved by the Laboratory Animal Welfare and Ethics Committee in Institute of Microbiology Chinese Academy of Sciences. Prophylactic and therapeutic efficacy studies in mice used 6- to 8-week-old female SPF BALB/c mice (Beijing Vital River Laboratory Animal Technology Co., Ltd.). All mice were allowed free access to water and standard chow diet and provided with a 12 h light and dark cycle (temperature: 20-25 °C, humidity: 40%-70%). All mice used in this study are in good health and are not involved in other experimental procedure. In the prophylactic studies, groups of five mice each received a dose of 1, 5, 10, or 30 mg/kg of PN-SIA28 or PBS buffer in a volume of 200 µL one day prior to intranasal challenge with 10 MLD_50_ of A/Shenzhen/TH002/2016 (H5N6) virus. In the therapeutic studies, groups of five mice each received 15 mg/kg of PN-SIA28 right after or 1, 2, or 3 days after challenge with 10 MLD_50_ of the A/Shenzhen/TH002/2016 (H5N6) virus. PBS buffer was administered at day 1 post-challenge. The survival rates and weight-loss statuses of the mice were monitored until 14 days after infection.

### Formation and purification of Fab or Fab/HA complexes

PN-SIA28 Fab was mixed with purified, His tag-depleted, recombinant H1, H14, and H18 HA trimers at a molar ratio of five parts Fab to one part HA to ensure saturation with Fab. The resulting PN-SIA28 Fab variable fragment-H1 HA (PN-SIA28/H1), PN-SIA28 Fab variable fragment-H14 HA (PN-SIA28/H14), and PN-SIA28 Fab variable fragment-H18 HA (PN-SIA28/H18) complexes were purified from unbound substrates by size-exclusion gel filtration chromatography (Superdex 200 10/300 column; GE Healthcare) in a buffer comprising 50 mM Tris-HCl (pH 8.0) and 150 mM NaCl or 20 mM Tris-HCl (pH 8.0) and 150 mM NaCl, respectively. PN-SIA28/H1, PN-SIA28/H14, and PN-SIA28/H18 complexes were eluted as single peaks between the 158 and 670 kDa molecular weight markers, and were concentrated to 10 mg/mL for subsequent crystallization studies.

### Structure determination of the PN-SIA28/HA complex

Crystallization was performed using the hanging drop vapor diffusion method at 18 °C by mixing an equal volume (0.1 mL) of the protein solution (10 mg/mL) and the reservoir solution. Preliminary crystallization conditions for the PN-SIA28/H1 complex were obtained after 7 days in several conditions, and diffraction quality crystals were obtained in 0.1 M magnesium acetate, 0.1 M sodium acetate, and 8% w/v PEG 8000 (pH 4.5). The PN-SIA28/H1 complex data set was collected from a single crystal at 3.2 Å resolution at the Shanghai Synchrotron Radiation Facility beamline 19U. The diffraction quality crystals for the PN-SIA28/H14 complex were obtained at 18 °C using the sitting drop method with 2.0-µL drops containing 10 mg/mL PN-SIA28/H14 in the no.29 condition of the Molecular Dimension Screening Kit (MD1-50, Box 2) consisting of 0.1 M Tris, 5 % w/v PGA-LM, and 20 % w/v PEG 3350. The PN-SIA28/H14 complex data set was collected from a single crystal at 3.4 Å resolution at the Shanghai Synchrotron Radiation Facility beamline 17U. The PN-SIA28/H18 complex was obtained at 2.6 Å in the no.39 condition of the Molecular Dimension Screening Kit (MD1-37, Box 2) consisting of 0.1 M Bis Tris and 25 % w/v PEG 3350 (pH 5.5) at the Shanghai Synchrotron Radiation Facility beamline 17U. The diffraction quality crystals of PN-SIA28 fab were obtained in 0.1 M HEPES (pH 7.0) and 12% PEG 3350. The data set was collected at 2.5 Å resolution at the Shanghai Synchrotron Radiation Facility beamline 17U.

Data collection and refinement statistics are presented in Supplementary Table 1. Data were processed and scaled using HKL2000^60^. The structures were solved by molecular replacement using Phaser^61^ from the CCP4 program suite^62^. Initial rigid body refinement was performed using REFMAC5^63^, and extensive model building was performed using COOT (version 0.8.9)^64^. Further rounds of refinement were performed using the phenix.refine program implemented in the PHENIX package (version 1.13-2998)^65^ with energy minimization, isotropic ADP refinement, and bulk solvent modelling. The structures were then adjusted using COOT and were refined with PHENIX. The stereochemistry and quality of the structural models was analyzed using Molprobity^66^, the PISA server^67^, and programs in the CCP4 suite and were validated using the wwPDB validation server (http://wwpdb-validation.wwpdb.org/validservice). All structures were generated using PyMOL (http://www.pymol.org).

### Reporting summary

Further information on research design is available in the Nature Portfolio Reporting Summary linked to this article.

## Supplementary information

Reporting Summary

Supplmentary Information

## Data Availability

The data that support this study are available from the corresponding author upon request. Atomic coordinates and structure factors of PN-SIA28 and the PN-SIA28/H1, PN-SIA28/H14, and PN-SIA28/H18 complexes generated in this study have been deposited to the Protein Data Bank (PDB) under the accession codes 8GV4, 8GV5, 8GV6 and 8GV7. Source data are provided with this paper.

## Source data

Source Data
