# Supplementary material for: Structural basis for a human broadly neutralizing influenza A hemagglutinin stem-specific antibody including H17/18 subtypes
Source: Nat Commun. 2022 Dec 9;13:7603. doi: 10.1038/s41467-022-35236-y (PMC9734383; doi:10.1038/s41467-022-35236-y)
Supplement: Supplementary file 2 — Supplmentary Information [file 41467_2022_35236_MOESM2_ESM.pdf]

Supplementary Information

Structural basis for a human broadly neutralizing influenza A hemagglutinin stem-specific antibody including H17/18 subtypes

Yulu Chen, et al

Yulu Chen, Fei Wang, Liwei Yin, Haihai Jiang, Xishan Lu, Yuhai Bi, Wei Zhang, Yi Shi, Roberto Burioni, Zhou Tong, Hao Song, Jianxun Qi, George F. Gao

Supplementary Tables 1-2

Supplementary Figures 1-7

Supplementary Table 1. Data collection and refinement statistics.

	**PN-SIA28**	**PN-SIA28/H1**	**PN-SIA28/H14**	**PN-SIA28/H18**
**Data collection**				
Space group	$P2_1$	$P6_3$	$P2_1$	$P6_3$
**Cell dimensions**				
a,b,c (Å)	76.6, 86.4, 82.9	85.8, 85.8, 231.3	91.7, 121.0, 129.8	140.4, 140.4, 195.7
α,β,γ(°)	90, 105.3, 90	90, 90, 120	90, 107.7, 90	90, 90, 120
Resolution (Å)	50-2.5 (2.50-2.59)*	50-3.2 (3.2-3.31)*	50-3.4 (3.40-3.52)	50.0-2.60 (2.60-2.69)
R_{sym} or R_{merge}	0.12 (0.39)	0.19 (2.40)	0.30 (1.30)	0.16 (1.68)
I/σI	15.4 (7.9)	15.3 (1.0)	5.58 (1.1)	19.83 (1.66)
Completeness (%)	97.9 (99.9)	73.2 (100)	87 (99.9)	94.1 (97.0)
Redundancy	6.2	12.6	5.4	16.5
No. of unique reflections	36196	15923	36855	66862
Wilson B value	26	46	53	37
**Refinement**				
Resolution (Å)	42-2.5 (2.50-2.59)*	45.6-3.2 (3.2-3.29)*	49.7-3.4 (3.40-3.49)	35.1-26.0 (2.60-2.69)
No. of reflections	36175	11635	32697	62969
R_{work} or R_{free}	18.2/22.3	25.3/31.5	21.9/26.4	20.0/21.9
**No. of atoms**				
Protein	6939	5507	15000	5670
Ligand/ion	0	14	126	141
Water	299	0	0	225
**B/factors**				

Protein	35	84	66	49
Ligand/ion	0	59	734	87
Water	35	0	0	47
**R.m.s.d**				
Bond lengths (Å)	0.003	0.004	0.003	0.005
Bond angles (°)	0.662	0.9	0.56	0.92

R.m.s.d., root mean squared deviation. *Values in parentheses are for highest-resolution shell.

Supplementary Table 2a. Interaction between PN-SIA28 and H1

Heavy chain		HA1 (H1)	contacts[a]	Total contacts[b]
CDRH3	F99	H38, T318, V40	28, 13, 2	(60)
	G100	H38, N20, H18	5, 3, 3	
	I100A	H18	6	
Heavy chain		HA2 (H1)	contacts[a]	Total contacts[b]
CDRH1	S31	I18	7	(121)
CDRH2	Y52A	I18, D19, G16 (1), M17, Y34	10, 7, 4, 1, 1	
	Y56	E150, Y34, A35, K153 (1)	3, 2, 3, 3	
CDRH3	I98	R49, I45	3, 4	
	F99	W21, I45, R49, I48	4, 1, 4, 2	
	G100	W21	1	
	I100A	I18, W21, G20, T41, I45	2, 17, 15, 1, 1	
	Y100B	I18 (1), D19	8, 4	
	I100C	D19, I45	3, 2	
	I100D	Q38	1	
	L100E	Q42, Q38	5, 1	
	N100F	R49	1	
Light chain		HA1 (H1)	contacts[a]	Total contacts[b]
FR3	S60	K280	1	(1)
Light chain		HA1 (H1)	contacts[a]	Total contacts[b]
CDR1	S31	D46	3	(55)
	W32	Q42, D46 (2), I45, R49	18, 14, 5, 7	
CDRL2	G50	R49	2	
	S52	E57	1	
	S53	N53	1	
CDR3	A50	Q42	1	
	H52	Q42	3	

a. Numbers represent the number of atom to atom contacts between the antibody residues and the HA residues, which were analyzed by the Contact program in CCP4 suite (the distance cutoff is 4.5 angstroms). Numbers in parentheses represent the number of hydrogen bonds between atoms.

b. Numbers in parentheses represent the total number of atom to atom contacts.

Supplementary Table 2b. Interaction between PN-SIA28 and H14

Heavy chain		HA1 (H14)	contacts[a]	Total contacts[b]
CDRH3	F99	S38, T318	7, 14	(28)
	G100	T318	3	
	I100A	S38	4	
**Heavy chain**		**HA2 (H14)**	**contacts[a]**	**Total contacts[b]**
CDRH2	Y52A	I18, D19, G16	11, 11, 1	(123)
	Y56	R153 (1), A35, E150	7, 3, 1	
CDRH3	I98	I45, W21	6, 1	
	F99	L52, W21, I48, I45, N49	3, 2, 7, 13, 12	
	G100	W21	2	
	I100A	W21, I18, G20, T41, I45	16, 5, 14, 1, 1	
	Y100B	I18 (1), D19	4, 2	
	I100C	D19, T41, I45	2, 1, 1	
	L100E	Q42, L38	2, 4	
**Light chain**		**HA2 (H14)**	**contacts[a]**	**Total contacts[b]**
CDRL1	S30	D46	1	(82)
	S31	D46	12	
	W32	Q42, D46(1), I45	28, 17, 4	
CDRL2	G50	N53	1	
	S52	N53 (1)	7	
	S53	N53 (1), N49	7, 1	
CDRL3	A91	Q42	1	

a. Numbers represent the number of atom to atom contacts between the antibody residues and the HA residues, which were analyzed by the Contact program in CCP4 suite (the distance cutoff is 4.5 angstroms). Numbers in parentheses represent the number of hydrogen bonds between atoms.

b. Numbers in parentheses represent the total number of atom to atom contacts.

Supplementary Table 2c. Interaction between PN-SIA28 and H18

Heavy chain		HA1 (H18)	contacts[a]	Total contacts[b]
CDRH3	F99	S38, T322	4, 5	(15)
	G100	H18	1	
	I100A	H18	5	
**Heavy chain**		**HA2 (H18)**	**contacts[a]**	**Total contacts[b]**
CDRH1	S31	I18	6	(131)
CDRH2	Y52A	D19, I18, G16, Y34	7, 13, 1, 2	
	D53	Y34	1	
	Y56	D150, K153(1), Y34, A35, H26	1, 4, 2, 3, 2	
	Y58	K38 (1)	5	
CDRH3	I98	V45	1	
	F99	W21, V45, T49, I48	12, 5, 3, 3	
	I100A	I18, G20, W21, T41, V45	4, 10, 11, 1, 1	
	Y100B	I18(1), D19	10, 11	
	I100C	D19, T41	5, 1	
	I100D	K38	1	
	L100E	Q42, K38	2, 3	
**Light chain**		**HA2 (H18)**	**contacts[a]**	**Total contacts[b]**
CDRL1	S30	D46 (1)	6	(64)
	S31	D46 (1)	5	
	W32	Q42, D46, V45	22, 10, 4	
CDRL3	A91	Q42 (1)	3	
	H92	Q42 (1)	12	
	F94	K38	2	

a. Numbers represent the number of atom to atom contacts between the antibody residues and the HA residues, which were analyzed by the Contact program in CCP4 suite (the distance cutoff is 4.5 angstroms). Numbers in parentheses represent the number of hydrogen bonds between atoms.

b. Numbers in parentheses represent the total number of atom to atom contacts.

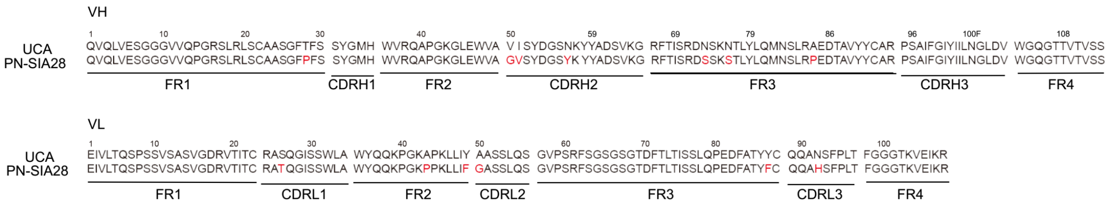

Supplementary Figure 1. Alignment of VH and VL amino acid sequences of PN-SIA28 and its unmutated common ancestor (UCA). The amino acid residues are numbered and the CDR and FR segments are labeled according to Kabat numbering. The amino acid substitutions are highlighted in red.

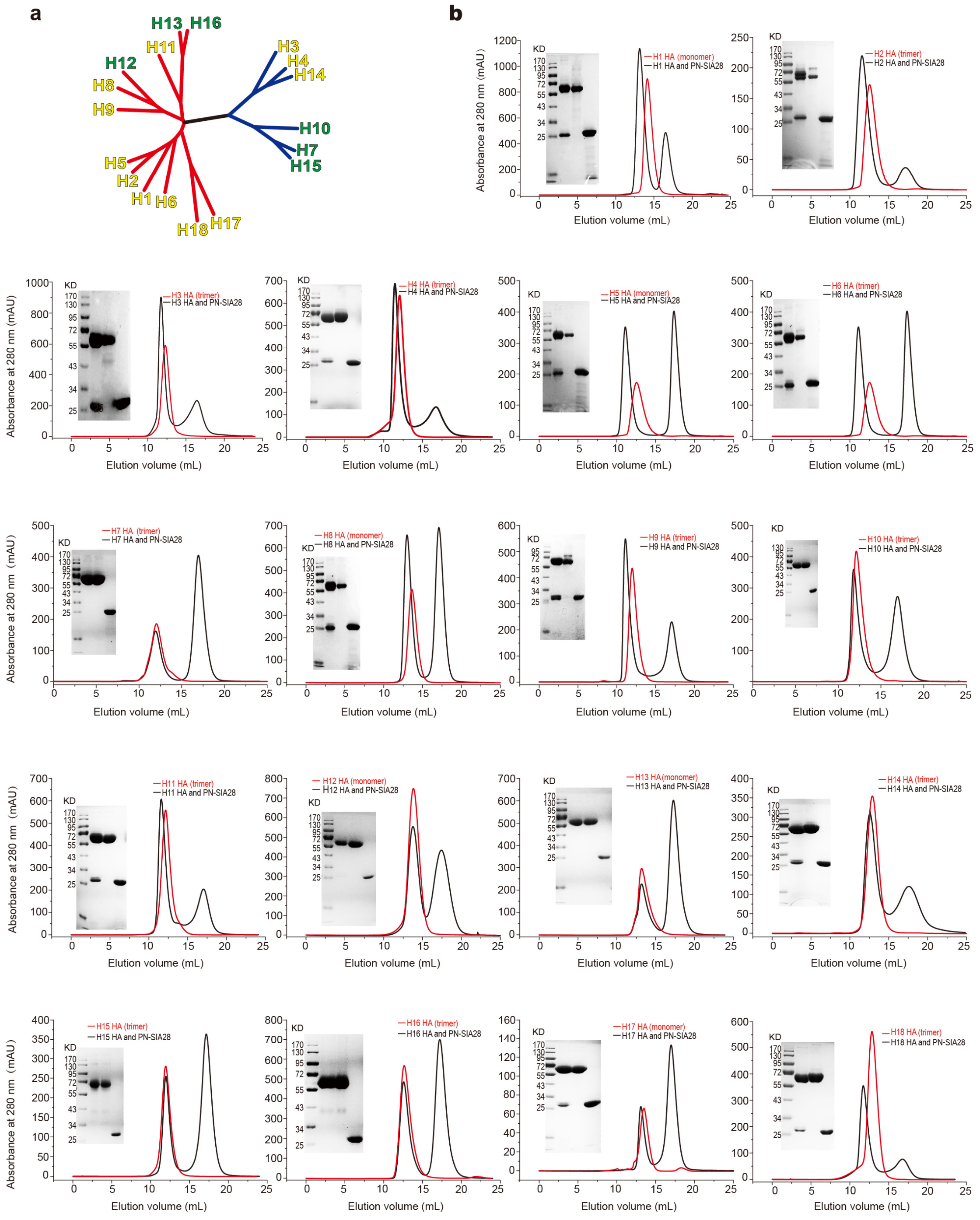

Supplementary Figure 2. Binding properties of different HA proteins with PN-SIA28.

a, Phylogenetic tree of influenza A HAs generated by MEGA software. Group 1 and group 2 are colored in red and blue. The HAs further subdivided into binding or not binding to PN-SIA28 are denoted in yellow and green characters, respectively. **b,** Gel filtration analysis of PN-SIA28. The HA proteins which bind to PN-SIA28 have a shifting forward elution peak in comparison to those HA proteins alone (H1, H2, H3,

H4, H5, H6, H8, H9, H11, H14, H17 and H18), whereas the HA proteins (H7, H10, H12, H13, H15 and H16) that cannot form complex with PN-SIA28 have no shift peak. The SDS-PAGE lanes represents the proteins of three peaks respectively. The data presented here are representative of two independent experiments with similar results. Source data are provided as a Source Data file.

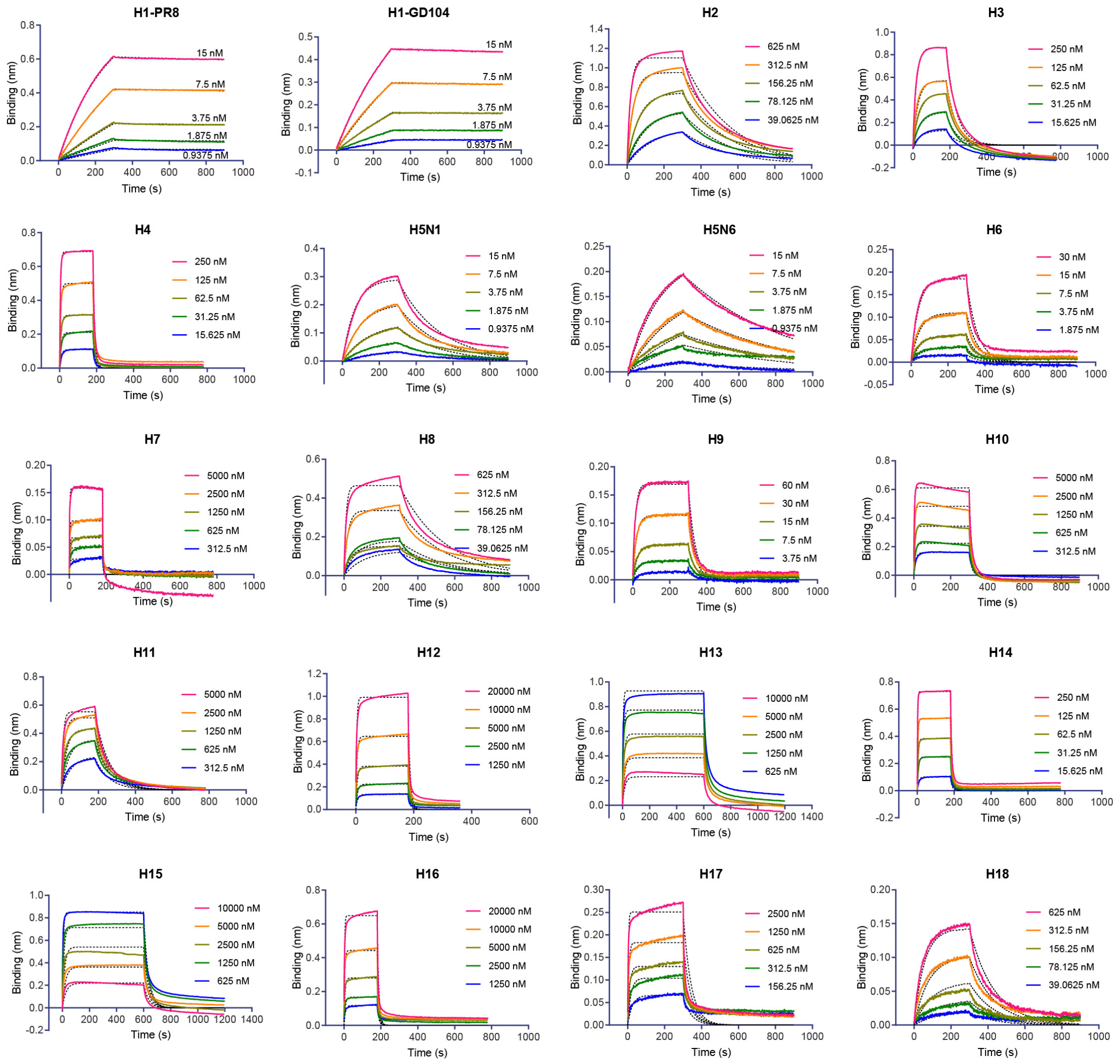

Supplementary Figure 3. Kinetic binding curves for interaction between different HAs and PN-SIA28. Solid curves are the experimental trace obtained from BLI experiments, and the black dotted curves were obtained by fitting data to the 1:1 binding model (Octet RED96 analysis software 7.0). Affinity measurements (KD values) for the binding curves were reported in Fig. 1a. The data presented here are representative of two independent experiments with similar results. Source data are provided as a Source Data file.

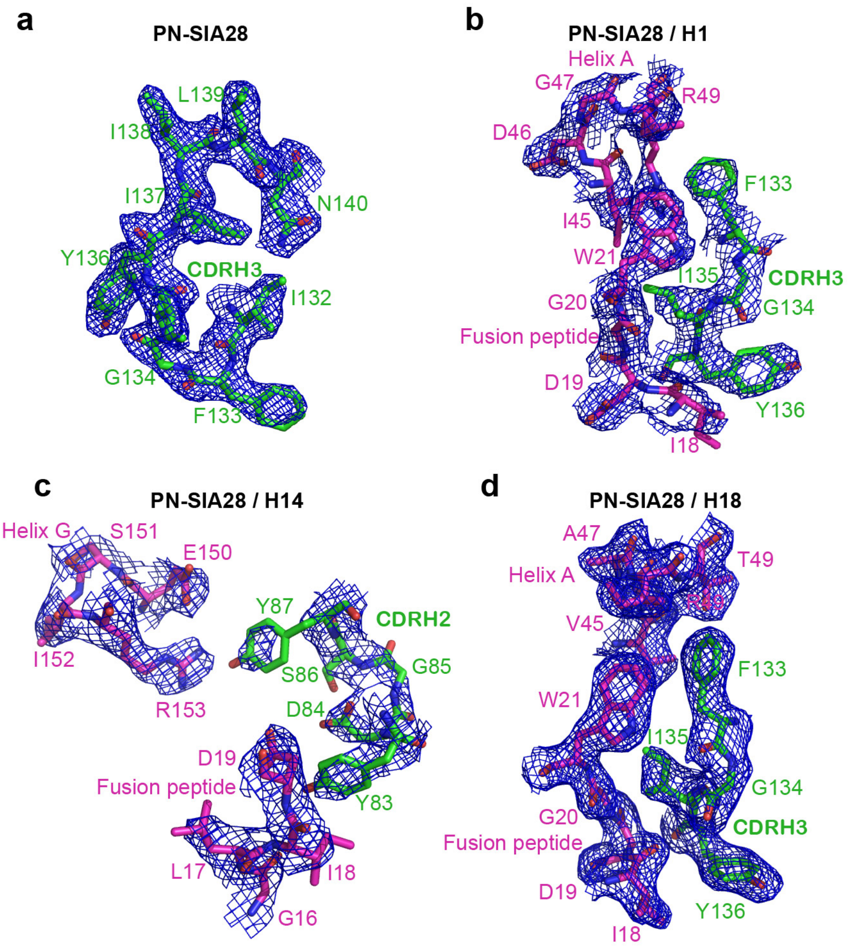

Supplementary Figure 4. Representative 2Fo-Fc maps showing a portion of PN-SIA28 or PN-SIA28/HA complex structures. The portion of 2Fo-Fc electron maps of PN-SIA28 (**a**), PN-SIA28/H1 (**b**), PN-SIA28/H14 (**c**), and PN-SIA28/H18 (**d**) structures are contoured at 1.0 σ level with the final structure shown in stick models. The 2Fo-Fc maps were generated by FFT program in CCP4 software, and the figures were drawn by Pymol software.

	HA1						HA2																				
Number / HA	18	20	38	40	280	318	16	17	18	19	20	21	26	34	35	38	41	42	45	46	48	49	52	53	57	150	153
H1	H	N	H	V	K	T	G	M	I	D	G	W	H	Y	A	Q	T	Q	I	D	I	R	V	N	E	E	K
H2	H	N	H	K	K	T	G	M	I	D	G	W	H	Y	A	K	T	Q	F	D	I	T	V	N	E	N	K
H5	H	N	H	Q	K	T	G	M	V	D	G	W	H	Y	A	K	T	Q	I	D	I	T	V	N	E	E	K
H6	H	N	H	V	T	T	G	L	I	D	G	W	H	Y	A	R	T	Q	I	D	I	T	V	N	E	E	K
H8	Q	N	Q	M	K	V	G	M	I	D	G	W	H	M	A	O	T	Q	I	D	I	T	V	N	E	E	K
H9	Q	T	H	K	K	V	G	L	V	A	G	W	H	M	A	R	T	Q	I	D	I	T	V	N	E	E	R
H11	L	N	S	V	K	T	G	L	I	N	G	W	H	I	A	K	T	Q	I	D	I	T	V	N	E	E	R
H17	Q	N	G	Q	K	T	G	M	I	D	G	W	H	Y	A	K	T	Q	V	D	I	T	V	N	E	D	K
H18	H	N	S	H	I	T	G	L	I	D	G	W	H	Y	A	K	T	Q	V	D	I	T	V	N	E	D	K
H12	Q	N	Q	E	E	I	G	L	I	A	G	W	H	I	A	R	T	Q	I	D	M	Q	L	N	E	D	R
H13	L	T	S	I	K	T	G	L	I	N	G	W	H	I	A	K	T	Q	I	D	I	T	I	N	E	E	R
H16	L	N	S	I	K	T	G	L	I	N	G	W	H	I	A	K	T	Q	I	N	I	T	I	N	E	E	R
H3	H	V	N	T	E	T	G	M	I	D	G	W	H	Q	A	L	T	Q	I	D	I	N	L	N	E	E	R
H4	H	V	T	Q	K	T	G	L	I	D	G	W	H	T	A	L	T	Q	I	D	I	N	L	N	E	E	R
H14	H	V	S	K	P	T	G	L	I	D	G	W	H	T	A	L	T	Q	I	D	I	N	L	N	E	E	R
H7	H	V	N	T	D	T	G	L	I	D	G	W	H	T	A	Y	T	Q	I	D	I	T	L	N	E	A	R
H10	H	V	N	T	K	T	G	M	V	D	G	W	H	Q	A	Y	T	Q	I	D	I	T	L	N	E	E	R
H15	H	V	N	T	E	L	G	L	I	D	G	W	H	T	A	Y	T	Q	I	D	I	T	L	N	E	E	R

Supplementary Figure 5. Sequence comparison of PN-SIA28 epitope among 18 HA subtypes. Hemagglutinin alignment showing the PN-SIA28 contact residues shaded in yellow as defined by residues within 4.5 Å of PN-SIA28.

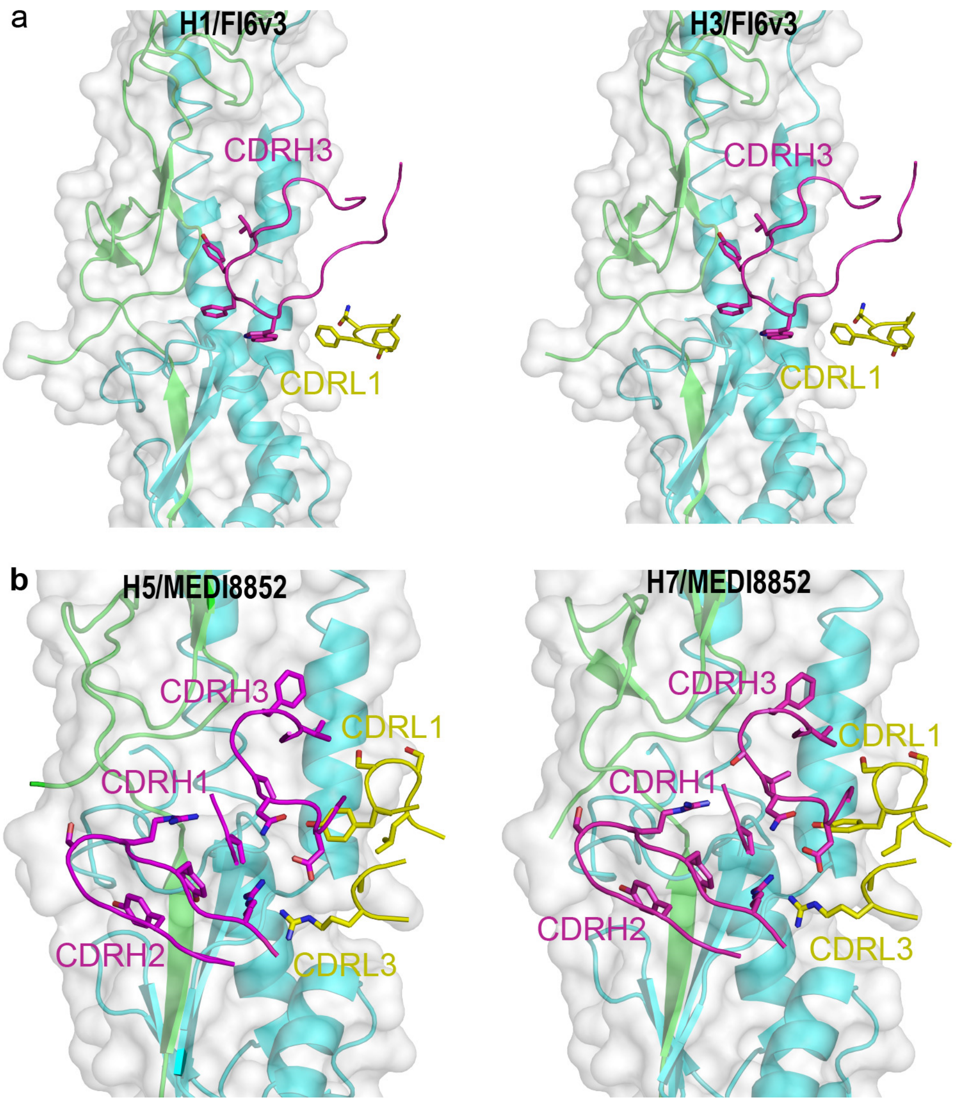

Supplementary Figure 6. Two bnAbs bind to HAs of different groups with same CDRs.

a-b, Two bnAbs interact with group 1 and group 2 HAs. The overall structure of the HA is shown in cartoon and surface representation from the side view. HA1 and HA2 of HA are colored in green and cyan, respectively. **a,** FI6v3 binds to H1 (group 1) and H3 (group 2) with the same CDRH (CDRH3) and CDRL (CDRL1). The CDRH and CDRL of FI6v3 are colored in magenta and yellow, respectively. The key interacting residues of FI6v3 are shown as sticks. **b,** MEDI8852 binds to H5 (group 1) and H7 (group 2) with the same CDRHs (CDRH1, CDRH2, CDRH3) and CDRLs (CDRL1, CDRL3). The CDRHs and CDRLs of MEDI8852 are colored in magenta and yellow, respectively. The key interacting residues of MEDI8852 are shown as sticks.

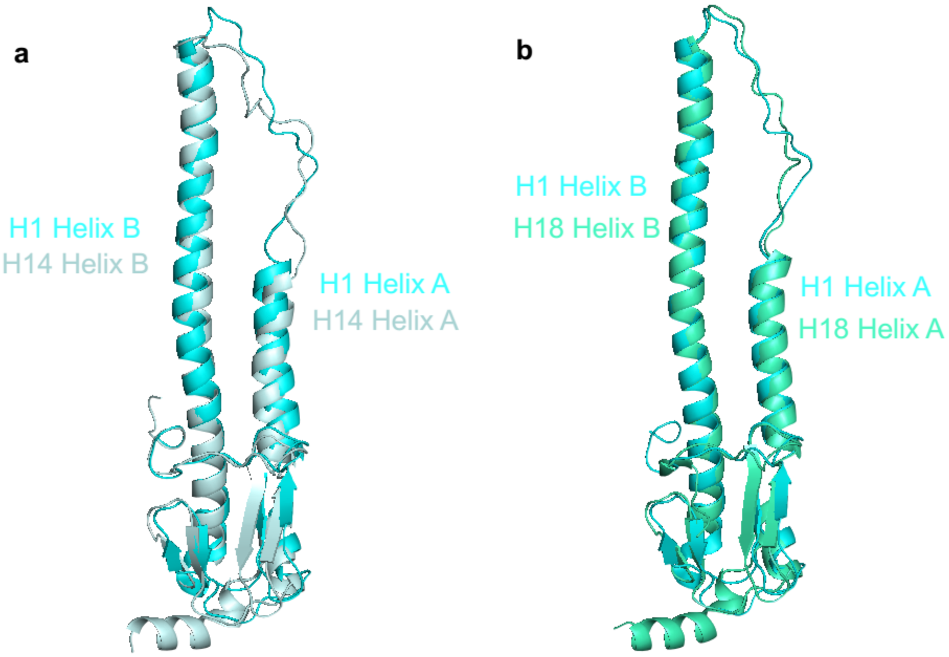

Supplementary Figure 7. Overlay of HA2s in HA/PN-SIA28 complexes. The overall structure of the HA2 is shown in cartoon representation from the side view. **a,** Overlay of HA2s in H1/PN-SIA28 and H14/PN-SIA28 complexes. The alignments were carried out by fixing Helix B. The HA2s are colored according to Fig. 4. **b,** Overlay of HA2s in H1/PN-SIA28 and H18/PN-SIA28 complexes. The alignments were carried out by fixing Helix B. The HA2s are colored according to Fig. 4**.**
